# Visualizing embryogenesis in the seed

**DOI:** 10.1093/plphys/kiae295

**Published:** 2024-05-22

**Authors:** Janlo M Robil, Dechang Cao

**Affiliations:** Assistant Features Editor, Plant Physiology, American Society of Plant Biologists; Department of Biology, School of Science and Engineering, Ateneo de Manila University, Quezon City 1108, Philippines; Assistant Features Editor, Plant Physiology, American Society of Plant Biologists; Germplasm Bank of Wild Species & Yunnan Key Laboratory for Crop Wild Relatives Omics, Kunming Institute of Botany, Chinese Academy of Sciences, Kunming, Yunnan 650201, China

An embryo is the product of union between the male and female gametes and allows life cycles to occur generation by generation. Generally, the body plan of plants and animals is established during embryogenesis. However, some seed plants take a pause during embryogenesis and thus have underdeveloped embryos in the seeds (Baskin and Baskin 2023). Underdeveloped embryos retard seed germination, called morphological dormancy, which is quite common in angiosperms (Baskin and Baskin 2014). Fossil records have also documented that tiny embryo-associated seed dormancy facilitated early angiosperms to successfully colonize the disturbance-prone planet (Marie Friis et al. 2015). Indeed, seeds of some extant gymnosperms (e.g. Cycadaceae, Ginkgoaceae, and Taxaceae) also show morphological dormancy (Baskin and Baskin 2014). Thus, the pause of embryogenesis and morphological dormancy of seeds contribute to a very successful strategy of plants to adapt to the diverse environments on the earth.

It has been challenging to monitor the embryogenesis process of seeds. Recently, x-ray micro-computed tomography (micro-CT) was developed to provide nondestructive structure imaging (Piovesan et al. 2021). Especially, the resolution of x-ray micro-CT imaging has been enhanced to examine cell–cell connection in small anatomic structures, such as xylem vessels (Camboué et al. 2024). In this issue of *Plant Physiology*, Ma et al. (2024) used X-ray micro-CT to generate high-resolution 3D imaging of *Ginkgo biloba* seeds at different developmental stages, providing a fine-scale trajectory of embryogenesis.

High-contrast staining is a crucial step of high-resolution x-ray micro-CT imaging. Ma et al. (2024) introduced cesium iodide (CsI) as an excellent staining agent to optimize the x-ray micro-CT imaging method. The optimized method allowed high-resolution 3D imaging. More than 2000 high-resolution virtual slices were collected for each *G. biloba* embryo sample. Subsequently, 3D reconstruction and morphological analyses provided detailed structural information to distinguish the tiny anatomical structures in the embryo (Ma et al. 2024). Freshly matured *G. biloba* seeds have tiny embryos consisting of cotyledons, hypocotyl, radicle, and the shoot apical meristem (SAM). Interestingly, Ma et al. (2024) also identified some unique structures such as long interconnected tubular structures and circular hollow structures, which are likely tracheids and secretory cavities, respectively (Fig.).

**Figure.**
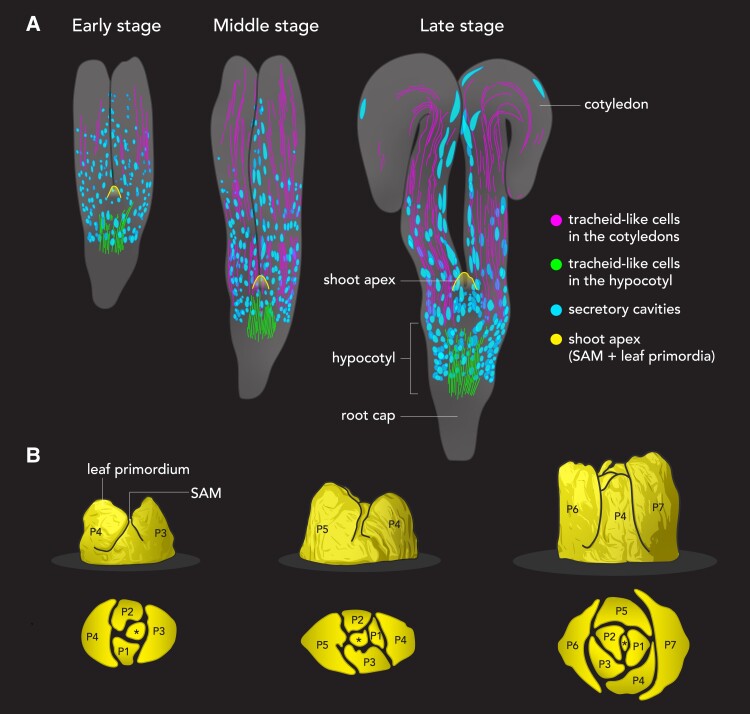
High-resolution x-ray micro-CT imaging reveals developmental trajectories of anatomical and morphological features in *G. biloba* embryos. **A)** A schematic diagram of different stages of *G. biloba* embryo maturation showing the distribution of tracheid-like cells (magenta and green) and secretory cavities (cyan). **B)** Shoot apex development during *G. biloba* embryo maturation. Top row: illustration of different stages of shoot apex development during embryo maturation of *G. biloba* based on 3D reconstruction images. Bottom row: a schematic diagram of the transverse section of the shoot apices at each stage showing the organization of the leaf primordia. Four leaf primordia were observed in the embryo of freshly collected seeds, which increased to seven in the fully matured embryo, as indicated by the plastochron (P) numbers. The asterisk indicates the SAM. Illustrations adapted from (Ma et al. 2024).

Consistent with previous reports (Wang et al. 2011), the *G. biloba* embryo continued to develop, with size increasing during storage after collection (Ma et al. 2024). When the stored seeds were examined 6 months after harvest, the cotyledons became fully expanded and exhibited convoluted tips, suggesting that the embryo had progressed to maturity (Fig. A). During the process of embryogenesis, the shoot apex continuously generated leaf primordia, and 7 leaf primordia were observed in the mature embryo. The leaf primordia were arranged in a spiral pattern around the SAM (Fig. B). Ma et al. (2024) obtained thin paraffin sections in parallel and observed similar developmental dynamics.

X-ray micro-CT images showed some long interconnected tubular structures in the *G. biloba* embryo. A comparison of x-ray micro-CT images and paraffin sections revealed that these microstructures were tracheids (Ma et al. 2024). 3D rendering of the x-ray micro-CT images revealed that the tracheids were distributed in the cotyledons and the hypocotyl. Surprisingly, the tracheids in these 2 tissues were not connected to each other throughout the embryogenesis, suggesting separate transport systems in the cotyledons and the embryo axis of *G. biloba* (Ma et al. 2024). The independent development of tracheids in the cotyledons and the hypocotyl of *G. biloba* was different from a previous report of *Arabidopsis thaliana*, in which the whole vascular system originates from a provascular cell initiated at the early globular stage (Yoshida et al. 2014). It is unclear whether such an independent development of tracheids also occurs in other gymnosperms.

The secretory cavity is another special structure in the *G. biloba* embryo. Some “hollow rings” found in the micro-CT transect sections of the embryo attracted special attention of the authors. Comprehensive analyses of the micro-CT images and serial tangential paraffin sections suggested that these “hollow rings” were cavity structures (Ma et al. 2024). Usually, cavity-like structures have been reported to secrete and accumulate secondary metabolites in leaves, flowers, and ovaries (Li et al. 2024). Ma et al. (2024) detected 520 metabolites in *G. biloba* embryo, including terpenes and flavonoids. However, it is not clear whether the metabolites were stored in the cavities.

In addition, Ma et al. (2024) analyzed gene expression patterns during embryogenesis of *G. biloba*. A total of 479 genes showed a gradual increase in mRNA abundance during the embryo development, which showed enriched Gene Ontology terms associated with vascular development and secretory cavities (Ma et al. 2024). Basic Local Alignment Search Tool analyses revealed that 93 genes are involved in regulating development, and 22 genes are associated with secretory cavity development in *G. biloba*. Further analyses of the expression patterns of these genes suggested that genes of the *PIN*, *ARF*, *CESA*, *CSLD*, *NAC*, *SHR*, *LBD*, and *MYB* families might be important regulators of vascular development in *G. biloba* embryo, and *bHLH*, *JAZ*, and *WRKY* might play major roles in the cavity development (Ma et al. 2024).

In summary, Ma et al. (2024) optimized the x-ray micro-CT imaging method to monitor the embryogenesis of *G. biloba* and identified some unique structures in the embryo. The independent development of tracheids in the cotyledons and the hypocotyl during embryogenesis of *G. biloba* provided new clues to investigate the evolution of vascular systems in plants. The cavities that were newly reported seed structures changed considerably in shape and size during embryo development of *G. biloba*, suggesting their possible roles in embryogenesis. Functions of these cavity structures are worthy of future research. The findings generated new insights into the embryogenesis of extant gymnosperms and also provided clues to mechanisms underlying morphological dormancy of seeds.
